# Array-Based DNA Methylation Profiling for Breast Cancer Subtype Discrimination

**DOI:** 10.1371/journal.pone.0012616

**Published:** 2010-09-07

**Authors:** Ilse Van der Auwera, Wayne Yu, Liping Suo, Leander Van Neste, Peter van Dam, Eric A. Van Marck, Patrick Pauwels, Peter B. Vermeulen, Luc Y. Dirix, Steven J. Van Laere

**Affiliations:** 1 Translational Cancer Research Group, Laboratory of Pathology, University of Antwerp/University Hospital Antwerp, Oncology Center, Sint-Augustinus, Antwerp, Belgium; 2 DNA Microarray Core, Sidney Kimmel Cancer Center, Johns Hopkins University, Baltimore, Maryland, United States of America; 3 Oncomethylome Sciences SA, Liège, Belgium; National Institute on Aging, United States of America

## Abstract

**Background:**

Abnormal DNA methylation is well established for breast cancer and contributes to its progression by silencing tumor suppressor genes. DNA methylation profiling platforms might provide an alternative approach to expression microarrays for accurate breast tumor subtyping. We sought to determine whether the distinction of the inflammatory breast cancer (IBC) phenotype from the non-IBC phenotype by transcriptomics could be sustained by methylomics.

**Methodology/Principal Findings:**

We performed methylation profiling on a cohort of IBC (N = 19) and non-IBC (N = 43) samples using the Illumina Infinium Methylation Assay. These results were correlated with gene expression profiles. Methylation values allowed separation of breast tumor samples into high and low methylation groups. This separation was significantly related to DNMT3B mRNA levels. The high methylation group was enriched for breast tumor samples from patients with distant metastasis and poor prognosis, as predicted by the 70-gene prognostic signature. Furthermore, this tumor group tended to be enriched for IBC samples (54% vs. 24%) and samples with a high genomic grade index (67% vs. 38%). A set of 16 CpG loci (14 genes) correctly classified 97% of samples into the low or high methylation group. Differentially methylated genes appeared to be mainly related to focal adhesion, cytokine-cytokine receptor interactions, Wnt signaling pathway, chemokine signaling pathways and metabolic processes. Comparison of IBC with non-IBC led to the identification of only four differentially methylated genes (*TJP3*, *MOGAT2*, *NTSR2* and *AGT*). A significant correlation between methylation values and gene expression was shown for 4,981 of 6,605 (75%) genes.

**Conclusions/Significance:**

A subset of clinical samples of breast cancer was characterized by high methylation levels, which coincided with increased DNMT3B expression. Furthermore, an association was observed with molecular signatures indicative of poor patient prognosis. The results of the current study also suggest that aberrant DNA methylation is not the main force driving the molecular biology of IBC.

## Introduction

Epigenetic changes, in particular DNA methylation, are recognized as one of the most common forms of molecular alteration in human cancer [1], [2]. Two changes in DNA methylation patterns are observed in cancer: i) a global hypomethylation, which has been associated with increased chromosomal instability, the reactivation of transposable elements and loss of imprinting; and ii) hypermethylation of CpG islands located in the promoter regions of tumor suppressor genes, which has conventionally been associated with transcriptional silencing in cancer [3], [4]. The DNA methylation patterns associated with the development and progression of cancer have potential clinical use [5]. First of all, the changes in DNA methylation patterns are characteristic of cancer cells, specific to the cancer type and occur at the early stages of cancer development, making them candidate biomarkers for early and specific cancer detection [6]. Second, DNA methylation patterns are of potential value for prognosis, as they might also reflect both the growth advantage and malignant potential of cancer cells. Third, changes in DNA methylation can affect genes influencing response to therapy, which makes these potential markers of clinical response.

Breast cancer is a heterogeneous disease that comes in several distinct clinical and histological phenotypes. Clinical progression is difficult to predict using the current prognostic factors and treatment is therefore not as effective as it should be. Genome-wide gene expression profiling by complementary DNA microarray has been used for accurate tumor subtyping based on a defined molecular signature [7], [8]. DNA methylation profiling might provide an alternative or complementary tactic to classify breast cancer and, in this way, provide clinicians with a better understanding of individual tumor biology and an opportunity to personalize patient treatment strategies. Previous studies have associated DNA methylation patterns with histological patterns of tumor growth [9], [10], histological tumor grade [11]–[13] and with hormone receptor and Her2/neu expression [12], [14]–[16]. Using an array-based platform with more than 800 cancer-related genes, Holm et al. have recently revealed that the molecular breast cancer subtypes, especially basal-like, luminal A and luminal B tumors, harbor specific methylation profiles [17].

In the present study, we undertook methylation profiling using the Infinium Human Methylation27 BeadChips (Illumina, San Diego, CA, USA) in a series of breast cancer cases to determine whether subsets of breast cancer can be distinguished by their profiles of methylation. In particular, we investigated the possibility that the inflammatory breast cancer (IBC) phenotype is characterized by a specific DNA methylation pattern. IBC is a particularly aggressive manifestation of primary epithelial breast tumors and affects ∼5% of women with breast cancer [18], [19]. Patients with IBC are often misdiagnosed due to the lack of knowledge about symptoms. Furthermore, no specific therapies have been developed for the treatment of IBC. Prognosis therefore remains dismal, with 5-year survival rates ranging from 30% to 50%. The absence of tailored treatment for IBC is due in part to a lack of understanding of the biological factors that influence the IBC disease course and outcome. Elucidating the molecular characteristics of IBC will help the development of novel diagnostic tools and innovative, targeted therapies for patients with IBC. Investigation of methylation in IBC has so far been restricted to studies focused on individual tumor suppressor genes, using quantitative methylation-specific PCR [20], [21]. These studies have reported differential methylation of certain genes between IBC and non-IBC, leading us to consider the possibility of a unique methylation profile for IBC.

## Methods

### Patients' samples

Breast tumor and normal breast tissue samples were retrieved from the tissue bank of the General Hospital Sint-Augustinus (Antwerp, Belgium). Clinical and pathological data are stored in a database in accordance with hospital privacy rules. Specimens were brought to the pathologists immediately after resection and part of the tissue was placed in liquid nitrogen and subsequently stored at −180°C. A total of 19 patients with IBC and 43 patients with non-IBC were included in this study. In addition, we collected 10 normal breast tissues from healthy controls (mean age 36y, range 25–47y). IBC was diagnosed according to the criteria mentioned in the AJCC (American Joint Committee on Cancer)-TNM staging system [22]. All patients with IBC showed diffuse enlargement of the involved breast of sudden onset. There was erythema and edema of the skin involving more than one third of the breast. The presence of dermal lymphatic invasion as an isolated observation was not sufficient for the diagnosis of IBC and was not necessary for the diagnosis either. Tumor characteristics are provided in Table 1.

**Table 1:** Tumor characteristics.

Clinicopathological features	IBC (N = 19)	Non-IBC (N = 43)	P-value
Patients' ages (y)			0.978
Mean	59,6	59,7	
Range	45–79	30–89	
Tumor stage			<0.001
I	0 (0%)	12 (28%)	
II	0 (0%)	16 (37%)	
III	12 (63%)	12 (28%)	
IV	7 (37%)	3 (7%)	
Histological tumor grade			0.049
Well	0 (0%)	9 (21%)	
Moderate	7 (37%)	18 (42%)	
Poor	12 (63%)	16 (37%)	
Estrogen receptor			0.608
Positive	12 (63%)	30 (70%)	
Negative	7 (37%)	13 (30%)	
Progesterone receptor			0.479
Positive	7 (37%)	20 (46%)	
Negative	12 (63%)	23 (54%)	
HER2 amplification			0.270
Positive	8 (42%)	12 (28%)	
Negative	11 (58%)	31 (72%)	

Ethical approval for collection of clinical material was obtained from the Sint-Augustinus Ethics Committee.

### Genomic DNA isolation and quality assessment

DNA extractions from fresh frozen tissues were performed using the QIAamp DNA Mini Kit (Qiagen, Valencia, Ca) according to the manufacturer's instructions. Genomic DNA quality was assessed by low agarose gel (0.5%) electrophoresis under low power voltage. Thresholds for genomic DNA quality check were: a) showing a high molecular band (∼40 Kb) in 0.6% agarose gel low-voltage electrophoresis (3 hrs) and no strong band of low molecular weight (<2.0 Kb); b) OD260/280 and OD260/230 within a range of 1.0–3.0.

### Bisulphite conversion

Bisulphite conversion of genomic DNA was done with the EZ DNA methylation Kit (Zymo Research, D5002) by following the manufacturer's protocol with modifications for the Illumina Infinium Methylation Assay. Briefly, one microgram of genomic DNA was first mixed with 5 µl of M-Dilution Buffer and incubated at 37°C for 15 minutes and then mixed with 100 µl of CT Conversion Reagent prepared as instructed in the protocol. Mixtures were incubated in a thermocycler for 16 cycles at 95°C for 30 seconds and 50°C for 60 minutes. Bisulphite-converted DNA samples were loaded onto 96-column plates provided in the kit for desulphonation and purification as instructed in the protocol. Concentration of eluted DNA was measured using a Nanodrop-1000. Bisulphite-converted samples were used for chip analysis as described below without any delay.

### Illumina Infinium Human Methylation27 BeadChip

Bisulphite-converted genomic DNA was analyzed using Illumina's Infinium Human Methylation27 Beadchip Kit (WG-311-1202) (performed at the DNA Microarray Core, Johns Hopkins University). Data can be freely downloaded from the web page http://www.tcrg.be/en/page6/page13/epigenetics.html. The beadchip contains 27,578 CpG loci covering more than 14,000 human RefSeq genes at single-nucleotide resolution. Chip process and data analysis were performed by using reagents provided in the kit and by following the manufacturer's manual. Briefly, 4 µl of bisulphite-converted genomic DNA was denatured in 0.014N sodium hydroxide, neutralized and amplified with kit-provided reagents and buffer for 20–24 hours at 37°C. Samples were fragmented. 12 µl of each sample was loaded onto a 12-sample chip and the chips were assembled into a hybridization chamber as instructed in the manual. After incubation at 48°C for 16–20 hours, chips were washed with wash buffers provided in the kit and assembled and placed into a fluid flow-through station for primer-extension reaction and staining with reagents and buffers provided in the kit. Polymer-coated chips were image-processed in Illumina's iScan scanner. Data were extracted using BeadStudio v3.0 software. Methylation values for each CpG locus are expressed as a beta (β)-value, representing a continuous measurement from 0 (completely unmethylated) to 1 (completely methylated). This value is based on following definition and calculation:

β-value  =  (signal intensity of methylation-detection probe)/(signal intensity of methylation- detection probe + signal intensity of non-methylation-detection probe).

As a positive control for methylation analysis, we used Human HCT116 methylated DNA (Cat# D5014-2, Zymo Research). As a negative control for methylation analysis, Human HCT116 DKO DNA (DNA methyltransferase double knock-out cells (DNMT1 and DNMT3b), prepared by Core Facility) was used. Replicate samples (N = 3) were included to assess inter-array reproducibility.

### Data analysis

Data were analyzed using Bioconductor in R (http://www.bioconductor.org). Sixty-eight CpG loci for which the detection p-value was >0.05 in 25% of samples were excluded from analysis as were 28 CpG loci showing a β-value of <0.5 in the Human HCT116 methylated DNA sample and 4,067 CpG loci showing a mean β-value of >0.2 in the Human HCT116 DKO DNA samples. Analysis was subsequently restricted to the remaining 23,496 CpG loci (12,956 genes), 18,742 located within CpG islands and 4,754 located outside of these regions.

Unsupervised hierarchical clustering analysis with the Euclidean distance and complete linkage algorithm was used to create a heatmap with associated dendrogram. A Prediction Analysis of Microarrays (PAM) and a two-sided t-test were used to identify differentially methylated CpG loci. Selection of the most significantly differentially methylated CpG loci between samples was based on (1) a β-value difference of >0.17 [23] and (2) a P-value of <0.0001. Functional characteristics of genes of interest were examined with a gene set enrichment analysis using hypergeometric testing for KEGG pathways.

### Gene expression profiling

Gene expression profiling of these breast tumors (N = 57) was performed as previously described [24]. Briefly, extracted RNA was hybridized onto the Affymetrix HGU133 plus 2.0 array in collaboration with the VIB Micrarray Facility (UZ-Gasthuisberg, Leuven, Belgium). Perfect match fluorescence intensities were background-corrected, mismatch-adjusted, normalized and summarized to yield log2-transformed gene expression data using the GCRMA algorithm. A pairwise Pearson correlation analysis was used to assess the association between methylation levels and gene expression levels.

## Results

### Unsupervised hierarchical clustering of normal breast tissues and breast tumors

**Figure 1:**
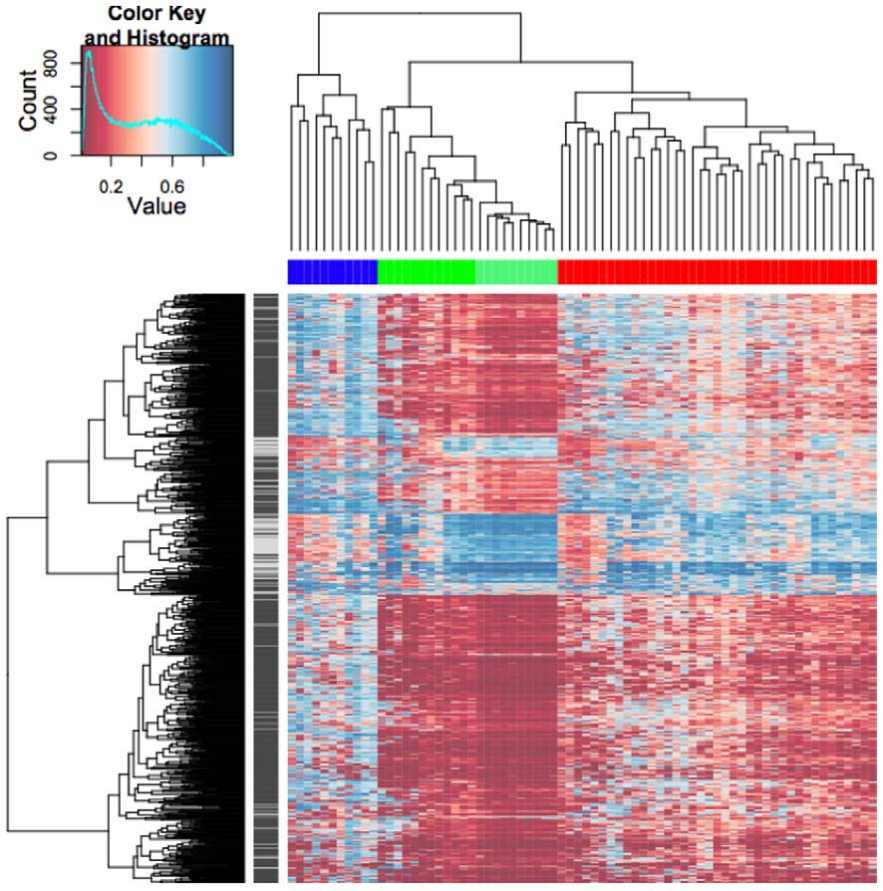
Hierarchical clustering of methylation values (β) from 1,000 CpG loci from 62 breast tumor and 10 normal breast tissue samples. Columns represent samples; rows represent CpG loci. Color represents methylation level β from 0 to 1 as per color bar (red  =  low methylation level; blue  =  high methylation level). Vertical color bar indicates location of CpG locus within the CpG island (dark grey) or outside of CpG island (grey). Top horizontal color bar indicates sample cluster. Samples separated into three distinct groups: a group consisting of 11 tumor samples (blue), a group consisting of 39 tumor samples (red) and a group including all normal breast tissue samples (light green) and 12 tumor samples (dark green).

**Figure 2:**
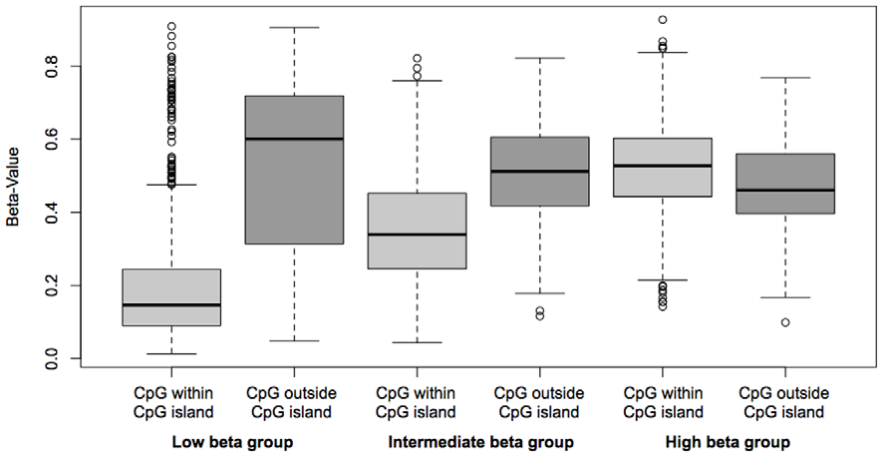
Box plots of methylation values (β) in the low, intermediate and high β groups according to the location of a CpG locus within a CpG island (light grey) or outside a CpG island (dark grey). Within the low and intermediate β groups, mean β-values for CpG loci outside a CpG island were significantly higher than mean β-values for CpG loci within a CpG island. Within the high β group, mean β-values for CpG loci outside a CpG island were significantly decreased.

DNA methylation levels were measured using the Illumina Infinium HumanMethylation27 BeadChips in breast tumor samples from 19 patients with IBC and 43 patients with non-IBC and in normal breast tissue samples from 10 healthy women. Unsupervised hierarchical cluster analysis of the 1,000 most varying β-values (largest s.d.) of the breast tumor (N = 62) and normal breast tissue samples (N = 10) separated samples into three distinct groups (average silhouette width 0.165, P<0.00001): one group consisting of 11 tumor samples (blue color bar in Figure 1), one group consisting of 39 tumor samples (red color bar in Figure 1) and one group that included all 10 normal samples and 12 tumor samples (green color bar in Figure 1). The normal breast tissue samples showed little variation in methylation profiles, with a mean s.d. of β-value of 0.02. The three sample clusters significantly differed in mean β-values (P<0.00001). The low β group (green color bar in Figure 1) had a mean β-value of 0.25, the intermediate β group (red color bar in Figure 1) had a mean β-value of 0.38 and the high β group (blue color bar in Figure 1) had a mean β-value of 0.52. Of the 1,000 most varying CpG loci, 835 were located within a CpG island (dark grey color bar in Figure 1) and 165 were located outside of a CpG island (grey color bar in Figure 1). Within the low and intermediate β groups, mean β-values for CpG loci outside a CpG island were significantly higher than mean β-values for CpG loci within a CpG island (P<0.00001) (Figure 2). In contrast, within the high β group, mean β-values for CpG loci outside a CpG island were significantly decreased (P<0.00001).

### Supervised analysis of methylation in breast tumors vs. normal breast tissues

To identify the CpGs showing the most significant tumor-specific changes in methylation relative to normal controls, a mean β-value was determined for all 62 breast tumor samples and compared with the corresponding mean β-value in the normal breast tissue group. Using the criteria of P<0.00001 and Δβ >0.17, 1,353 CpG loci (corresponding to 1,134 genes) were identified (Table S1). For 1,037 of these CpG loci (77%) a significant increase in methylation was observed in the group of breast tumors. Thus, 316 CpG loci showed significant loss of methylation in breast tumors relative to normal breast tissues. CpG loci outside of CpG islands were significantly over-represented in the group showing loss of methylation in breast tumors (76% versus 18%; P<0.0001, Fisher's exact test). To determine the biological relevance of the differentially methylated genes, we performed a gene set enrichment analysis using hypergeometric testing for KEGG pathways. Genes differentially methylated between normal breast tissues and breast tumors were related to focal adhesion (P<0.0001), extracellular matrix receptor interaction (P = 0.0025), pathways in cancer (P = 0.0049), cytokine-cytokine receptor interaction (P = 0.0066) and ether lipid metabolism (P = 0.0099) (Table 2).

**Table 2:** Biological function of genes differentially methylated between normal breast tissues and breast tumors.

KEGG pathway	KEGG id	Genes	P-value
Focal adhesion	04510	*CD1D, CD8A, CD9, IL11RA, THPO, ITGA4, EPO, CD33, TPO, CD34, IL1A, FCGR1A, KIT, CSF2, CD37*	<0.0001
ECM receptor interaction	04512	*FLT4, PDGFRB, LAMA1, CCND1, LAMA2, COL11A2, RELN, ACTN2, PARVG, COL1A2, BCL2, LAMA4, COL5A2, ITGA4, CCND2, PAK7, COL1A2, PGF, PPP1CC, COL6A3, FLT1, COL6A2, ITGA8, MYLK, EGFR, THBS4, CCND2, COL5A3, COL11A1, PRKCG*	0.0025
Pathways in cancer	05200	*LAMA1, LAMA2, COL11A2, RELN, COL1A2, SV2A, LAMA4, COL5A2, ITGA4, COL6A3, COL6A2, ITGA8, THBS4, COL5A3, COL11A1*	0.0049
Cytokine-cytokine receptor interaction	04060	*FLT4, LEP, TNFRSF10D, CX3CL1, IL11RA, PF4V1, TNFRSF10D, TNFRSF1B, ACVR1, TNFRSF18, CD40, CCL1, CXCL6, CCL18, TNFRSF8, EPO, TNFSF11, TPO, CCL7, FLT1, EGFR, CCL13, IL23A, INHBC, IL1A, TNFSF8, KIT, CSF2, TGFBR1, XCL1*	0.0066
Ether lipid metabolism	00565	*AKR1B1*	0.0099

### Unsupervised hierarchical clustering of breast tumors

**Figure 3:**
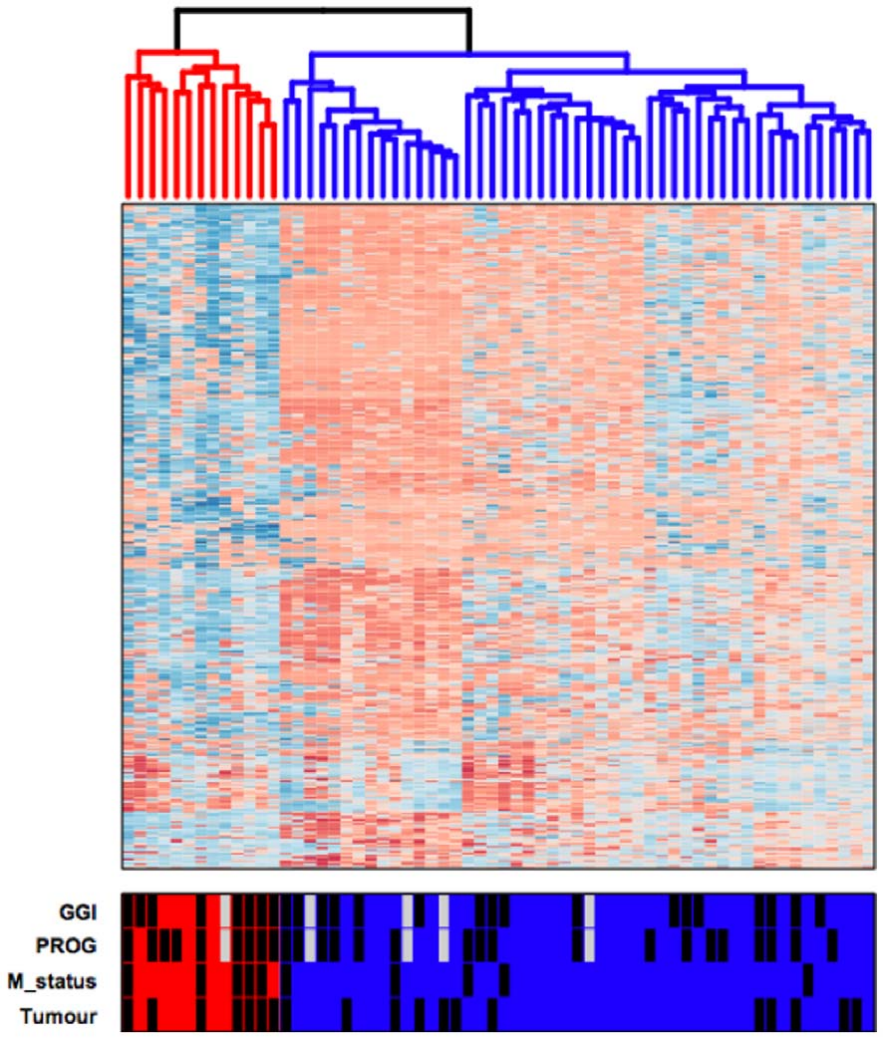
Hierarchical clustering of methylation values (β) from 500 CpG loci from 62 breast tumor samples. Columns represent samples; rows represent CpG loci. Color represents methylation level β from 0 to 1 as per color bar (red  =  low methylation level; blue  =  high methylation level). Samples separated into two distinct groups: a high β group consisting of 13 breast tumor samples (red dendrogram) and a low β group consisting of 49 breast tumor samples (blue dendrogram). Bottom horizontal bar indicates the distribution of samples according to the genomic grade index of Sotirou et al. [26] (black fill  =  grade 3, no fill  =  grade 1, grey fill  =  unknown), the 70-gene prognostic signature of van 't Veer et al. [25] (black fill  =  poor prognosis, no fill  =  good prognosis, grey fill  =  unknown), M status (black fill  =  positive, no fill  =  negative) and tumor subtype (black fill  =  IBC, no fill  =  non-IBC).

Unsupervised hierarchical cluster analysis of 500 most varying β-values (largest s.d.) separated breast tumor samples into two distinct groups (average silhouette width 0.235, P<0.0001) (Figure 3). We refer to these clusters as low β (N = 49) and high β (N = 13) tumor groups. These groups showed a mean β-value of 0.314 and 0.513, respectively (P<0.0001). The high β tumor group was significantly enriched for breast tumor samples from patients with distant metastasis and poor prognosis (as determined by the 70-gene prognostic signature [25]) when compared to the low β tumor group (P χ2 = 0.026 and P χ2 = 0.049, respectively). Furthermore, the high β tumor group tended to be enriched for IBC samples (54% and 24% of samples in the high and low β tumor groups, respectively, were from IBC patients, P χ2 = 0.087) and samples with a high genomic grade index [26] (67% and 38% of samples in the high and low β tumor groups, respectively, had a high genomic grade index, P χ2 = 0.100). There was no difference between the two tumor groups with regard to age, tumor stage, histological grade or ER, PR and HER2 expression.

To investigate whether the difference in methylation profiles between breast tumors is related to differences in the DNA methyltransferase (DNMT) machinery, we compared the expression levels of DNMT1, DNMT3a and DNMT3b mRNAs between the high β and low β groups of breast tumors (Figure S1). We observed increased mRNA expression levels of DNMT3b in the high β group of breast tumors (P = 0.034), with a mean expression level of 5.76 in the low β tumor group and 6.52 in the high β tumor group. Also for DNMT1, higher mRNA expression levels were observed in the high β tumor group, but this difference did not reach statistical significance (P = 0.088). No difference in DNMT3a expression between the two groups of breast tumors was measured (P = 0.620). The difference in DNMT3b expression between the high β and low β groups of breast tumors became far more modest when the mRNA expression levels were normalized according to those of the cell proliferation marker PCNA (P = 0.060).

We used Prediction Analysis of Microarrays (PAM) to classify low β and high β breast tumors by their methylation profile. PAM builds a classifier based on a ranking of CpG loci using a penalized t-statistic and then determines the misclassification error rate through 10-fold cross-validation. The optimal classifier included 16 CpG loci (corresponding to 14 genes) (Figures S2 and S3). Genes in the classifier were *NIP*, *CHGA*, *OSR1*, *GFRA3*, *KLK10*, *SSTR1*, *EFCPB2*, *PPARG*, *PRKAR1B*, *ABCG2*, *FGF5*, *PLTP*, *GRASP* and *PAX7*. Adding more genes to the classifier increased the error rate, whereas fewer genes did not have enough power to discriminate between classes. This CpG loci set showed excellent performance, classifying 47 of 49 low β and 13 of 13 high β tumor samples correctly for an overall success rate of 97%.

### Biological relevance of differentially methylated genes in high vs. low β breast tumors

Next, to identify differentially methylated genes between the high β group of breast tumors (N = 13) and the low β group of breast tumors (N = 49), we compared the mean β-values in both groups for each CpG locus. We then determined their biological relevance by performing a gene set enrichment analysis using hypergeometric testing for KEGG pathways. Using the criteria of P<0.0001 and Δβ >0.17, this analysis resulted in the identification of 450 CpG loci (corresponding to 366 genes) (Table S2). Genes differentially methylated between the low β and the high β group of breast tumors appeared to be mainly related to focal adhesion (P = 0.006), galactose metabolism (P = 0.0106), cytokine-cytokine receptor interaction (P = 0.0126), Wnt signaling pathway (P = 0.0221), fructose and mannose metabolism (P = 0.0289), chemokine signaling pathway (P = 0.0332) and pyruvate metabolism (P = 0.0407) (Table 3).

**Table 3:** Biological function of genes differentially methylated between the low β and the high β group of breast tumors.

KEGG pathway	KEGG id	Genes	P-value
Focal adhesion	04150	*COL6A2, CCND2, COL1A2, COMP, MYL9, SHC3, FLT1, COL11A2*	0.0060
Galactose metabolism	00052	*AKR1B1, PFKP*	0.0106
Cytokine-cytokine receptor interaction	04060	*TNFRSF10D, KIT, CXCL5, CNTFR, CXCL12, CXCL2, CXCL6, CXCL3, CX3CL1, FLT1*	0.0126
Wnt signaling pathway	04310	*CCND2, SFRP5, SFRP1, TCF7, SFRP2, FZD2*	0.0221
Fructose and mannose metabolism	00051	*AKR1B1, PFKP*	0.0289
Chemokine signaling pathway	04062	*CXCL5, ADCY4, LYN, CXCL12, CXCL2, GNG4, CXCL6, SHC3, CXCL3, CX3CL1*	0.0332
Pyruvate metabolism	00620	*LDHB, AKR1B1, ACOT12*	0.0407

### Supervised analysis of methylation in IBC versus non-IBC

To identify genes differentially methylated between IBC and non-IBC, we compared the mean β- value in IBC with the mean β-value in non-IBC for each CpG locus. Using the criteria of P<0.0001 and Δβ>0.17, only four CpG loci (corresponding to four genes) were identified. For all CpG loci, methylation values were increased in IBC in comparison to non-IBC. These included *TJP3* (tight junction protein-3), *MOGAT2* (monoacylglycerol O-acyltransferase 2), *NTSR2* (neurotensin receptor 2) and *AGT* (angiotensinogen).

### Reproducibility and correlation of array results with qMSP results

There was an excellent inter-array correlation between replicate DNA samples obtained from two breast tumors and a Human HCT116 DKO DNA sample (mean *r* ^2^ = 0.974; range 0.948–0.992).

**Figure 4:**
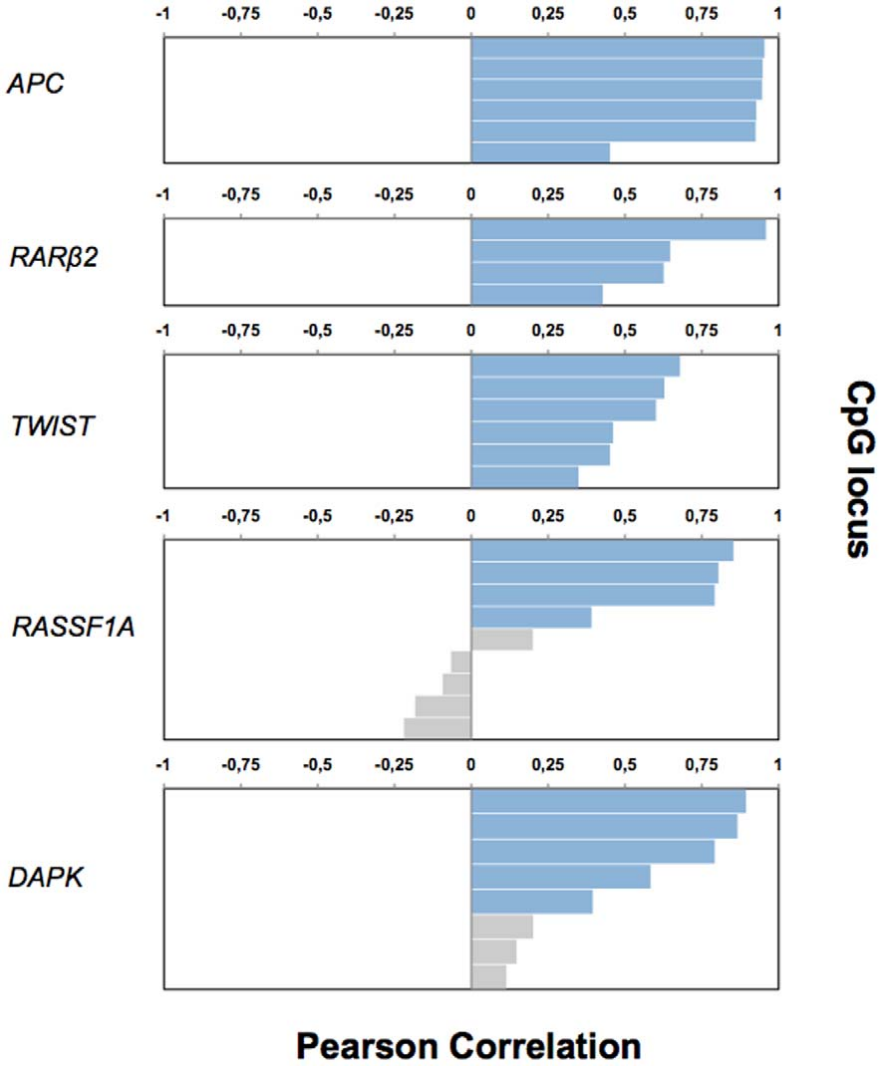
Analysis of correlation between methylation values from qMSP and the Infinium methylation array for five genes in 60 breast tumor samples. These five genes were represented by 33 CpG loci on the Infinium methylation array (y-axis). Pearson correlation values between methylation values from qMSP and the Infinium methylation array are shown on the x-axis, with negative values representing inverse correlations and positive values representing positive correlations. Significant correlation (P<0.01) are indicated in blue.

We compared quantitative methylation values studied by the Infinium methylation array with previously established methylation values by qMSP in 60 breast tumor samples [20]. This was done for five genes (*APC*, *RASSF1A*, *TWIST*, *RARβ2* and *DAPK*), which corresponded to 33 CpG loci on the Infinium methylation array. For 25 CpG loci, we observed significant positive correlations between methylation values by the Infinium methylation array and by qMSP (Figure 4).

### Correlation with gene expression

**Figure 5:**
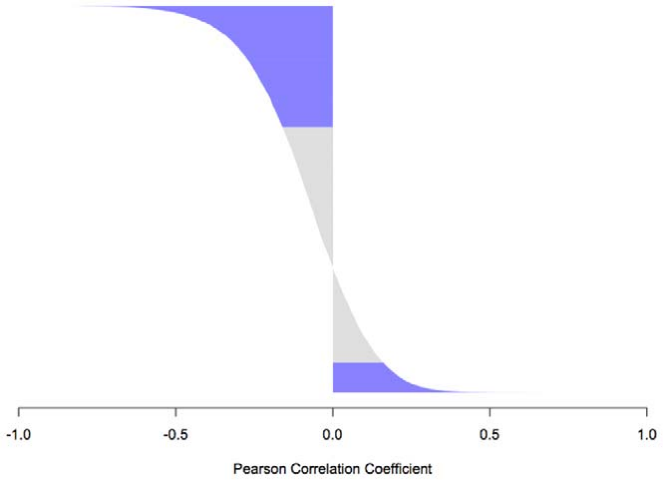
Analysis of correlation between methylation level and gene expression in 57 breast tumor samples. Pearson correlation values between methylation level and mRNA expression level are shown on the x-axis, with negative values representing inverse correlations and positive values representing positive correlations. Significant correlations (P<0.004, FDR<0.01) are indicated in blue.

We were able to correlate methylation results with gene expression profiles for 6,605 unique genes in 57 breast tumor samples. We performed a Pearson correlation analysis to evaluate correlations between methylation levels from 12,400 CpG loci and gene expression data from 10,494 probe sets (corresponding to these 6,605 genes). This analysis resulted in 19,884 correlation coefficients (range: (−0.83)−(+0.65)) (Figure 5). A significant (P<0.004, FDR<0.01) inverse correlation was observed for 6,229 of the correlated pairs and a significant positive correlation was observed for 1,534 of the correlated pairs. Overall, a significant correlation (either positive or negative) between methylation results and gene expression level was observed for 4,981 of 6,605 genes (75%).

## Discussion

High throughput methylation profiling platforms such as the Illumina Infinium methylation assay enable extensive methylation profiling of human tumors for a large number of genes. In the present study, we used this approach to assess the methylation profiles in a set of breast tumors and normal breast tissues. Unsupervised hierarchical cluster analysis of methylation values for the 1,000 most varying CpG loci (based on s.d.) identified three distinct groups for which mean methylation values significantly differed. Sample segregation was based primarily on the gain of methylation within CpG islands and the loss of methylation outside CpG islands relative to normal breast tissues. This is supportive of the basic theory that CpGs located within CpG islands in normal cells are unmethylated, whereas CpGs located outside CpG islands are methylated with the inverse pattern occurring in tumor cells [4]. A number of genes with higher methylation levels in tumor samples than in normal tissue samples proved to be involved in cancer pathways. We did not observe a perfect separation of normal breast tissue samples from breast tumors as a number of breast tumor samples clustered together with the normal breast tissue samples. Since we used whole tumor samples, this finding might be due to a confounding effect of non-neoplastic tissue on the methylation level measured in these samples.

The term ‘CpG island methylator phenotype’ or ‘CIMP’ was first used to describe a distinct subset of colorectal tumors that display high rates of concordant methylation of specific genes [27]. Subsequently, a similar phenotype has been described for a wide range of neoplasms including tumors of the ovary [28], bladder [29], prostate [29], stomach [30], liver [31], pancreas [32], esophagus [33] and kidney [34], as well as melanoma [35], neuroblastomas [36], leukemias [37] and lymphomas [38]. In some tumor types, such as hepatocellular carcinoma, melanoma, neuroblastoma or leukemia, CIMP has been shown to be associated with disease progression or poor patient survival [35], [36], [39], [40]. In colorectal cancer, the role of CIMP in prognosis depends on the microsatellite instability screening status [41]. In particular, the CIMP-high and microsatellite stable tumors show a poor prognosis. Evidence for a CIMP phenotype among breast cancer only recently emerged from a methylation profiling study analyzing a set of breast cancer cell lines [42]. The authors observed concurrent methylation-dependent silencing of a number of genes in breast cancer cell lines expressing a hypermethylator phenotype. Moreover, the hypermethylation defect in these breast cancer cell lines was related to aberrant overexpression of DNMT3b. These observations are in agreement with our data in clinical samples of breast cancer. In this study, unsupervised hierarchical cluster analysis of the methylation values of 500 CpG loci revealed two groups of breast tumors that possess different methylation signatures: high methylation and low methylation breast tumors. A set of 16 CpG loci (14 genes) correctly classified 97% of samples into the low or high methylation group of breast tumors. The high methylation group of breast tumors was more frequently associated with poor prognosis, as determined by the 70-gene prognostic signature of van 't Veer et al. [25]. Moreover, these breast tumors showed increased DNMT3b mRNA levels. These observations combine to suggest that a subset of breast tumors could display a CIMP, although this needs to be validated in an independent data set. Supervised analysis of the low and high methylation group of breast tumors revealed several differentially methylated genes implicated in different biological processes such as focal adhesion, cytokine-cytokine receptor signaling, chemokine signaling pathway, Wnt signaling pathway and metabolic processes. Interestingly, some of these genes have been previously associated with CIMP in other tumor types, such as *p73*, *GSTP1*, *SOCS-1*, *CACNA1G*, *CRABP1*, *NEUROG1* and RUNX3 [39], [43], [44]. Moreover, for other highly methylated genes, loss due to epigenetic silencing has been previously implicated in aggressive tumor biology. For example, methylation of the Wnt antagonists *SFRP5* and *SFRP1* in breast cancer is an independent risk factor for adverse patients survival [45], [46]. The CXCL12 chemokine binds to the CXCR4 receptor and contributes to survival, proliferation, and migration of malignant cells. Breast cancer cells lacking expression of CXCL12 but exhibiting CXCR4 can metastasize to target organs that secrete CXCL12 [47]. Epigenetic silencing of *CXCL12* has been shown to increase the metastatic potential of mammary carcinoma cells [48]. CpG hypermethylation of *COL1A2* has been shown to contribute to proliferation and migration activity of human bladder cancer [49]. The promoter methylation status of *CCND2* is associated with poor prognosis in human epithelial ovarian cancer [50].

We were interested to compare the methylation profiles of IBC and non-IBC, as little information is available on this topic. At the global level, we did not find evidence for a discriminating methylation profile. IBC samples did seem to be overrepresented in the group of tumors showing high methylation values, but this observation did not reach statistical significance and thus needs to be further investigated on a larger sample population. For only four genes (*TJP3*, *MOGAT2*, *NTSR2* and *AGT*), methylation values were significantly higher in IBC than in non-IBC. TJP3 functions in maintaining tight junction integrity and in transducing regulatory signaling events in patients with primary breast cancer [51]. Loss of tight junction plaque molecules in breast cancer is associated with a poor prognosis. MOGAT2 is involved in dietary fat absorption from the small intestine. NTSR2 belongs to the G protein-coupled receptor family and binds the ligand neurotensin. Several reports implicate neurotensin in numerous detrimental functions linked to neoplastic progression of several cancer types, including pancreatic, prostate, colon and lung cancers [52]. AGT is involved in the suppression of tumor growth and metastasis [53], [54]. The overexpression of human AGT decreases angiogenesis and prevents tumor sinusoids from remodeling and arterialization, thus delaying tumor progression in vivo [54]. Interestingly, several studies have indicated that, compared with non-IBC samples, IBC samples show increased angiogenesis. Histologically, increased vascular density and high fractions of proliferating endothelial cells have been observed in clinical IBC samples [19], [55]. Using qRT-PCR, we demonstrated that mRNA levels of several angiogenic growth factors and their receptors were higher in clinical IBC samples when compared to non-IBC samples [56]. In two previous studies that focused on methylation of individual tumor suppressor genes in IBC, we observed increased methylation frequencies for two genes, *APC* and *RARβ2*, by using quantitative methylation-specific PCR [20], [21]. Also in this study, higher methylation levels for these genes were measured in IBC samples, but this difference did not meet our selection criteria for differential methylation.

We observed a high level of correlation between methylation and expression levels. Using the GoldenGate Methylation Cancer Panel I from Illumina, O'Riain et al. observed a significant correlation between methylation values and reduced gene expression in follicular lymphoma for up to 28% of CpG loci [57]. Holm et al. recently studied correlations between methylation status and gene expression in breast cancer by using a similar technique [17]. They reported that a highly significant fraction (72%) of the expression-methylation pairs showed inverse correlation between relative methylation levels and expression levels. Thus, these results are very similar to ours.

In summary, this study suggests the existence of a CIMP in a subset of clinical samples of breast cancer. Breast tumors displaying a CIMP also showed increased expression of DNMT3b. Further studies are necessary to elucidate the mechanisms underlying this phenotype and to demonstrate the potential clinicopathological implications of a CIMP in breast cancer. Patients with breast cancer displaying a CIMP might benefit significantly from a targeted demethylation treatment as an adjunct to standard chemotherapeutic regimens. The results of the current study also suggest that aberrant DNA methylation is not the main force driving the molecular biology of IBC. More research needs to be done to fully understand the biological factors that influence the IBC disease course and outcome.

## Supporting Information

Figure S1 Box plots of mRNA expression levels for DNMT3B and DNMT1 in the high β and low β groups of breast tumors. In the high β group of breast tumors, higher mRNA expression levels for DNMT3B and DNMT1 were observed in comparison to the low β group of breast tumors.
(0.09 MB TIF)

Figure S2 Results of PAM analysis. The 62 breast tumor samples (x-axis) are plotted against the probabilities to belong to either class high β (green) or low β (red). For each sample, two small circles are plotted: the red one showing the probability that this sample belongs to the low β group of breast tumors and the green one that it belongs to the high β group of breast tumors. The classifier correctly predicted 47 of 49 low β and 13 of 13 high β samples for an overall success rate of 97%.
(0.12 MB TIF)

Figure S3 Box plots of methylation levels in the low β and high β groups of breast tumors for the 16 CpG loci belonging to the classifier identified by PAM analysis.
(0.14 MB TIF)

Table S1 (0.24 MB XLS)

Table S2 (0.09 MB XLS)
