# Supplementary figures and images for: Array-Based DNA Methylation Profiling for Breast Cancer Subtype Discrimination
Source: PLoS One. 2010 Sep 7;5(9):e12616. doi: 10.1371/journal.pone.0012616 (PMC2935385; doi:10.1371/journal.pone.0012616)

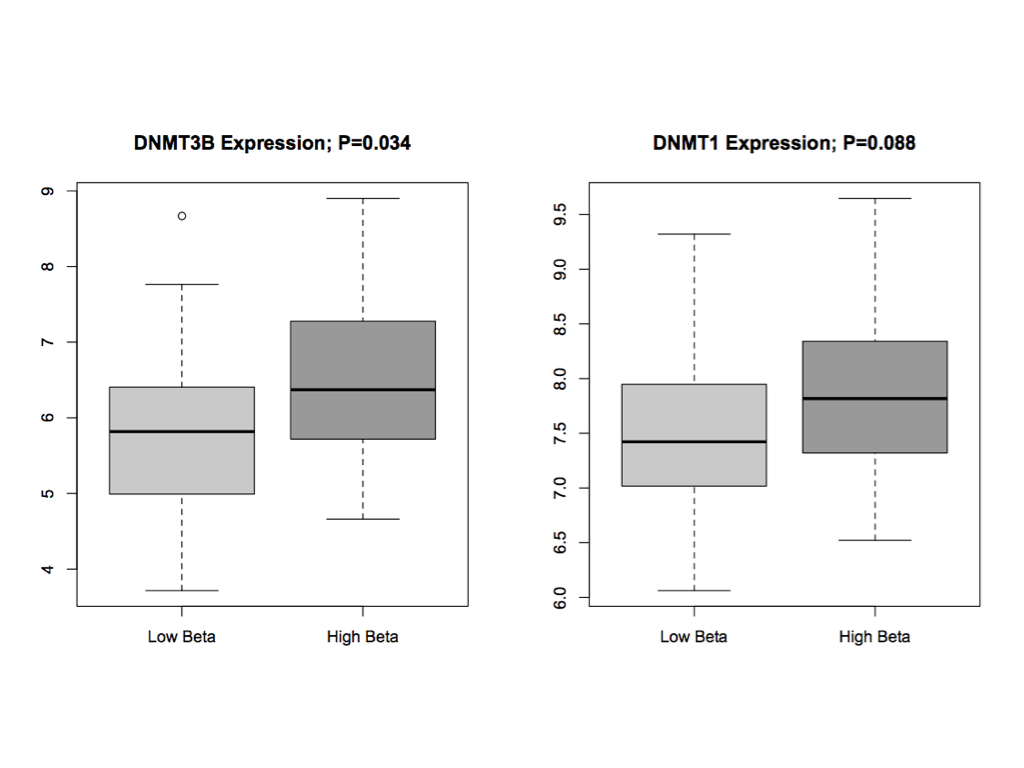

Supplement: Figure S1 — Box plots of mRNA expression levels for DNMT3B and DNMT1 in the high β and low β groups of breast tumors. In the high β group of breast tumors, higher mRNA expression levels for DNMT3B and DNMT1 were observed in comparison to the low β group of breast tumors. (0.09 MB TIF) [file pone.0012616.s001.tif]

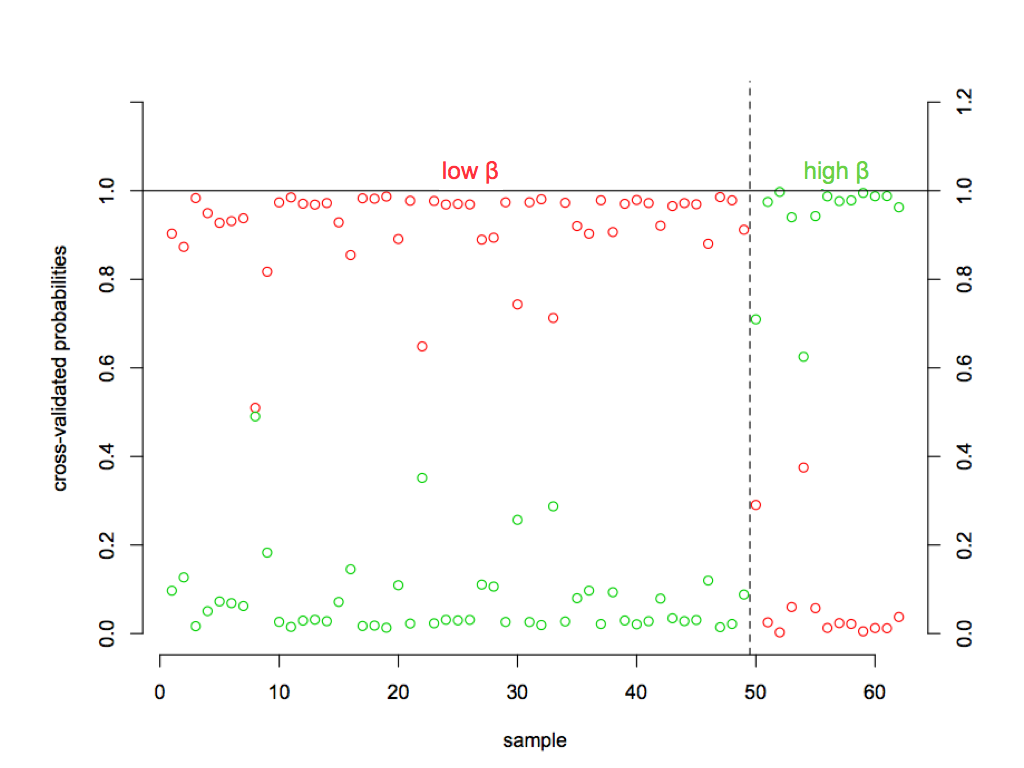

Supplement: Figure S2 — Results of PAM analysis. The 62 breast tumor samples (x-axis) are plotted against the probabilities to belong to either class high β (green) or low β (red). For each sample, two small circles are plotted: the red one showing the probability that this sample belongs to the low β group of breast tumors and the green one that it belongs to the high β group of breast tumors. The classifier correctly predicted 47 of 49 low β and 13 of 13 high β samples for an overall success rate of 97%. (0.12 MB TIF) [file pone.0012616.s002.tif]

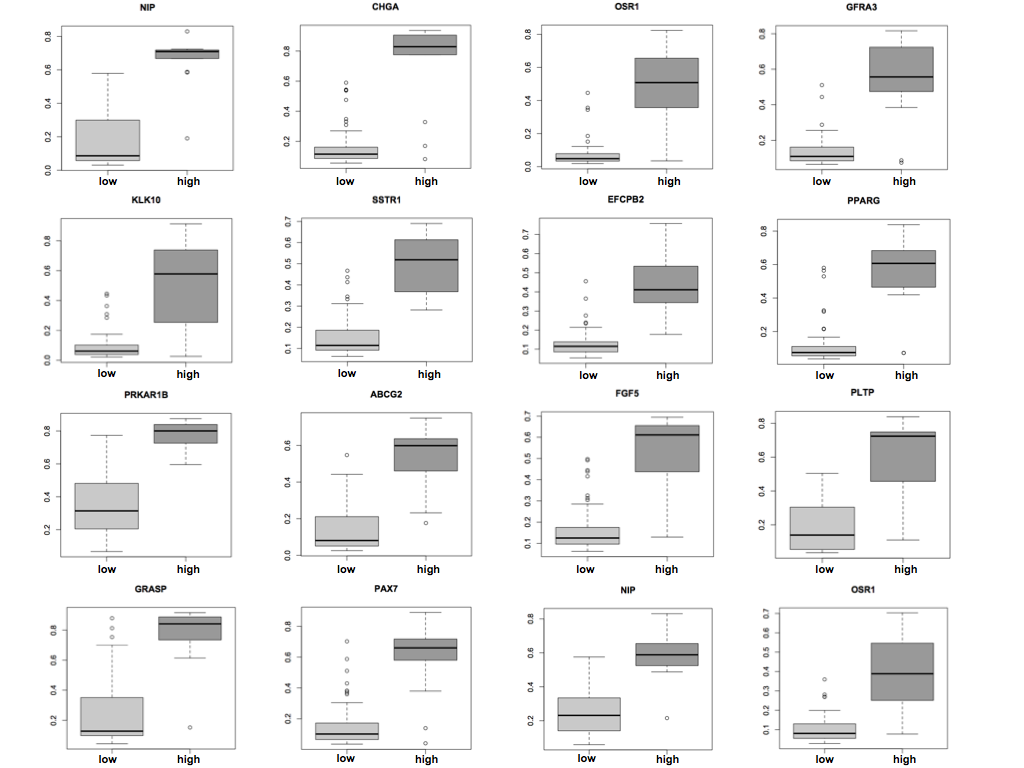

Supplement: Figure S3 — Box plots of methylation levels in the low β and high β groups of breast tumors for the 16 CpG loci belonging to the classifier identified by PAM analysis. (0.14 MB TIF) [file pone.0012616.s003.tif]
